# Transcriptome screen for fast evolving genes by Inter-Specific Selective Hybridization (ISSH)

**DOI:** 10.1186/1471-2164-11-126

**Published:** 2010-02-22

**Authors:** Juan I Montoya-Burgos, Aurélia Foulon, Ilham Bahechar

**Affiliations:** 1 Department of Zoology and Animal Biology, University of Geneva, 30 quai Ernest Ansermet, 1211 Geneva 4, Switzerland

## Abstract

**Background:**

Fast evolving genes are targets of an increasing panel of biological studies, from cancer research to population genetics and species specific adaptations. Yet, their identification and isolation are still laborious, particularly for non-model organisms. We developed a method, named the Inter-Specific Selective Hybridization (ISSH) method, for generating cDNA libraries enriched in fast evolving genes. It utilizes transcripts of homologous tissues of distinct yet related species. Experimental hybridization conditions are monitored in order to discard transcripts that do not find their homologous counterparts in the two species sets as well as transcripts that display a strong complementarity between the two species. Only heteroduplexes that disanneal at low stringency are used for constructing the resulting cDNA library.

**Results:**

We demonstrate the efficiency of the ISSH method by generating a brain cDNA library enriched in fast evolving transcripts of a non-model catfish species as well as a control, non-enriched library. Our results indicate that the enriched library contains effectively more fast evolving sequences than the control library. Gene annotation analyses also indicate enrichment in genes with low expression levels and non-ubiquitously expressed genes, both categories encompassing the majority of fast evolving genes. Furthermore, most of the identified transcripts show higher sequence divergence between two closely related catfish species as compared to recognized fast evolving DNA markers.

**Conclusions:**

The ISSH method offers a simple, inexpensive and efficient way to screen the transcriptome for isolating fast evolving genes. This method opens new opportunities in the investigation of biological mechanisms that include fast evolving genes, such as the evolution of lineage specific processes and traits responsible for species adaptation to their environment.

## Background

Fast evolving DNA sequences are used for answering a broad range of biological questions relative to population processes and phylogeography [e.g. [1]], species diversification [e.g. [2,3]], conservation biology [4] and also genome or phenotype mapping [e.g. [5]]. However, due to the very same intrinsic quality for which they are looked for, i.e. their high evolutionary rate, fast evolving DNA sequences display "lineage specific" changes and therefore require de novo development each time a new group of non-model organisms is being investigated. Despite various methodologies targeted toward the isolation of unspecific polymorphic DNA fragments [e.g. [6-8]] the identification and the isolation of fast evolving DNA sequences in non-model organisms is still laborious and expensive, making it a major impediment to the routine analysis of multiple loci on many taxa.

The isolation of fast evolving genes has gained new motivation and attention as genes involved in several actively investigated processes display high substitution rates: the evolution of species specific traits such as the human brain [e.g. [9,10]], speciation genes [e.g. [11,12]], reproduction genes [e.g. [13,14]] or genes governing the evolution of adaptive traits [e.g. [15]]. Theoretical approaches suggest that adaptation genes should be fast evolving so that selection could have a substrate on which to act [16]. Furthermore, speciation genes, those that are directly or indirectly involved in the establishment of the genetic barrier between closely related species, consistently displayed high divergence rates [11]. At present, fast evolving genes which often evolve under positive selection can be identified either through large genomic comparisons which are feasible only for model organisms like *Drosophila *species [e.g. [17,18]] or human-chimpanzee comparisons [19] or via long term experimental approaches such as in the discovery of the hybrid inviability gene *Hmr *in *Drosophila *[20]. The increasing interest in biological mechanisms driven by fast evolving genes appeals to the development of a more efficient and cost effective method for the isolation of such genes across closely related species and which would not imply the prior knowledge of genetic or genomic information.

**Figure 1:**
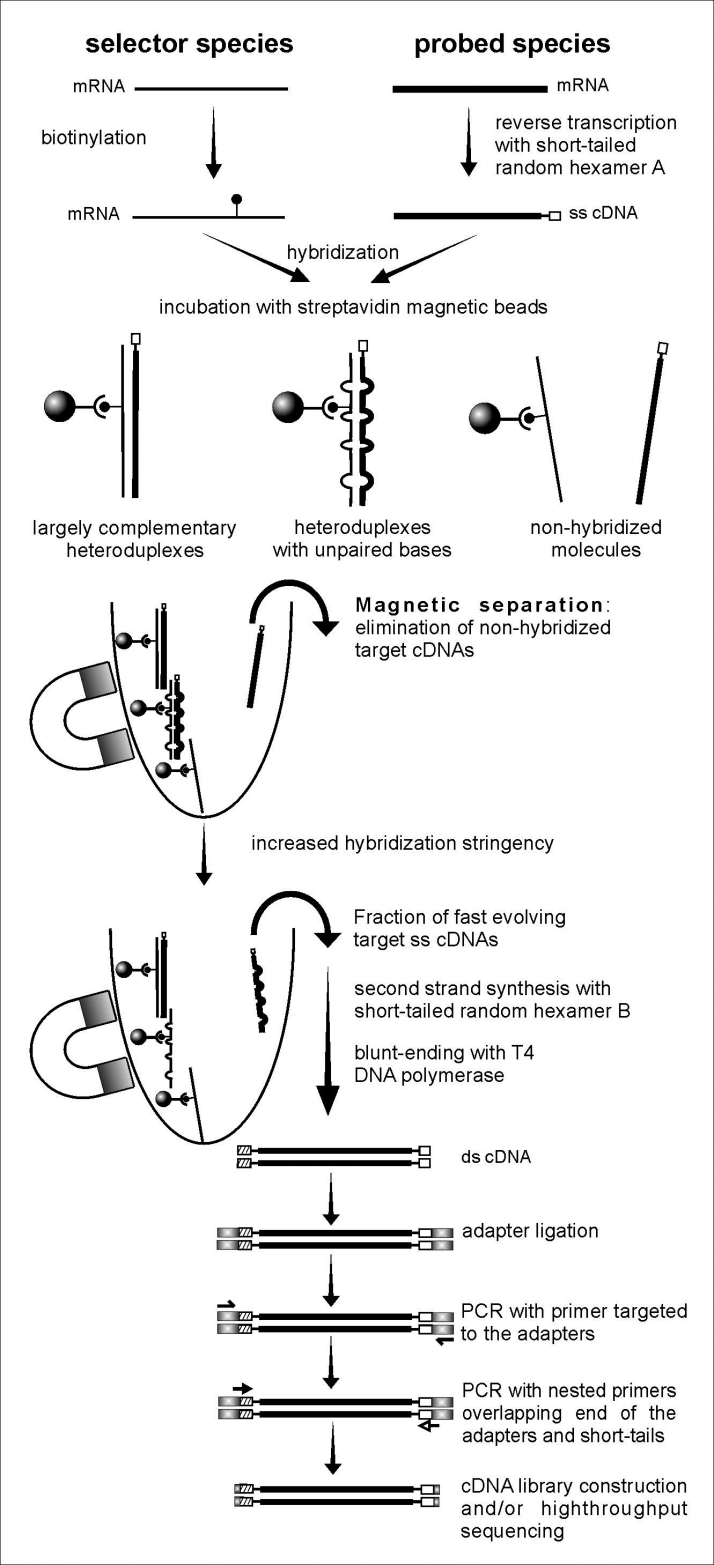
**Schematic representation of the ISSH method**. The cDNA pool of the species of interest, whose fast evolving transcripts are to be isolated, is called the "probed" while the mRNA pool of the species used as a template is called the "selector". Thick lines, probed transcripts; thin lines, selector mRNAs; small black dot, biotin; small opened or dashed bars at the donor transcript ends, tails of the short-tailed random primers A and B; grey ball, magnetic beads coated with streptavidin; magnet shape, magnetic separator; grey bars at the ends of short-tailed random primers, double strand adapters; arrows, PCR primers. Fast evolving transcripts which are isolated with the ISSH method are shown at the bottom of the chart.

Here we describe a simple and efficient experimental approach for enriching a cDNA library in fast evolving transcript fragments. Our method, named Inter-Specific Selective Hybridization (ISSH) is based primarily on the principles of the widely used subtractive hybridization (SH) procedure developed more than two decades ago for isolating cDNAs of differentially expressed genes [21-23]. In the original SH protocol, the hybridization of cDNAs versus mRNAs of different cell lines belonging to the same organism distinguishes transcripts that are equally expressed in both cell lines from those that are uniquely or differentially expressed in the cell line of interest. In the ISSH approach (Figure 1), the transcriptome of the species of interest ("probed" species) is reverse transcribed into single stranded (ss) cDNA and then hybridized against the biotinylated mRNA pool of a distinct yet close relative ("selector" species). During hybridization, three populations of transcripts of the species of interest can be found: (1) transcripts that never find their selector counterparts due to differential expression or gene loss; (2) fast evolving transcripts that find their homologous counterparts but the resulting heteroduplexes are unstable due to numerous nucleotide mispairings; and (3) conserved transcripts that form stable heteroduplexes. The second population of transcripts is in fact composed by sequences of varying divergence. The transcripts of interest are those that disanneal at the lowest stringencies and are therefore rescued for constructing the cDNA library enriched in fast evolving transcripts. The ISSH method was applied for isolating fast evolving transcript fragments of the non-model catfish species *Ancistrus temminckii *(family Loricariidae). We used the zebrafish genome as a reference for assessing the sequence divergence of the isolated transcripts and for transcript annotation and characterization.

## Results

### Experimental design

The ISSH method (Figure 1) confronts in solution complementary transcriptomes of two closely related species with the aim of rescuing transcripts of fast evolving genes. The property of evolving fast implies that such transcripts will disanneal at low stringencies from the heteroduplexes formed by homologous complementary sequences of the two species. Our method was applied to build a cDNA library enriched in fast evolving transcript fragments of brain tissue of the catfish *Ancistrus temminckii*. We used as the selector species its close relative *Ancistrus dolichopterus*. To assess the efficiency of the ISSH method we prepared a non-enriched control cDNA library of brain tissue of *A. temminckii *using standard protocols. The two libraries were sequenced with the FLX Genome Sequencer technology (Roche). We then "blasted" the enriched and control libraries against the complete genome of the zebrafish and analyzed the differences. We also annotated the transcripts producing significant matches and examined their characteristics to highlight the effectiveness of our method. As the zebrafish is not a close relative to our catfish and because the sequences of interest display high sequence divergence, a substantial proportion of the enriched library yielded no significant Blast matches. Therefore, we prepared an EST library of a close catfish relative, *Hypostomus *gr. *plecostomus*, belonging to the same subfamily (Loricariidae: Hypostominae), for refining the analyses.

### Analysis of sequence divergence

High-throughput sequencing and reads assembly yielded 2429 and 1255 contigs for the enriched and control libraries respectively. We blasted the contigs against the zebrafish genome using parameters suitable for comparing divergent sequences. Only Blast results with E-values lower than 10e-5 were considered for the analyses; they represented 45.7% and 40.6% of all contigs of the enriched and control libraries, respectively. Among the contigs producing non-significant Blast matches about half were low-complexity sequences as indicated by RepeatMasker and were excluded from further analyses (20.7% and 22% of the enriched and control libraries, respectively). Significant Blast alignments were classified by size in order to produce a finer analysis of the differences that exist between the enriched and control libraries. The sequence divergence comparisons (Table 1) consistently show that the enriched library displays more divergent sequences (higher mean) than the control library using as reference the zebrafish genome. Applying a mean t-test unambiguously indicates that the mean divergence per category is significantly higher in the enriched library than in the control library, and this for all size categories (Table 1).

**Table 1:** Analysis of sequence divergence for the enriched and the control libraries.

Size category	Enriched library		Control library		T-test (df-t)	*P*
(bp.)	Mean (S.D.)	n	Mean (S.D.)	n		
Blast against zebrafish						
90-109	29.93 (7.56)	126	26.93 (8.77)	66	-2.358 (116)	0.010*
110-129	33.32 (9.26)	129	28.84 (10.06)	67	-3.038 (124)	0.001**
130-149	34.82 (9.86)	138	31.61 (11.06)	61	-1.95 (103)	0.027*
150-169	36.84 (9.83)	103	33.56 (11.43)	47	-1.701 (78)	0.046*
170-189	40.42 (9.88)	100	33.34 (14.11)	36	-2.777 (47)	0.004**
190-209	40.97 (10.01)	94	37.03 (13.14)	39	-1.681 (57)	0.049*
210-229	40.52 (10.92)	80	28.91 (14.08)	55	-5.143 (96)	< 0.001**
230-249	39.33 (11.01)	64	34.63 (13.87)	34	-1.71 (55)	0.045*
≥ 250	42.39 (11.41)	145	34.72 (15.60)	34	-2.702 (41)	0.005**

Blast against *Hypostomus *catfish						
90-109	30.09 (9.93)	131	24.70 (12.86)	66	-2.986 (105)	0.002**
110-129	31.66 (11.29)	140	22.12 (12.62)	49	-4.771 (78)	< 0.001**
130-149	35.30 (12.17)	127	27.48 (12.30)	33	-3.261 (49)	< 0.001**
150-169	36.18 (12.55)	115	29.40 (15.87)	39	-2.423 (55)	0.009**
170-189	37.93 (11.71)	96	31.87 (13.66)	19	-1.807 (23)	0.04*
190-209	36.53 (12.15)	103	27.73 (12.41)	25	-3.193 (36)	0.001**
210-229	43.66 (12.01)	48	28.76 (15.48)	18	-3.689 (25)	< 0.001**
≥ 230	36.16 (12.08)	99	28.44 (18.10)	18	-1.740 (19)	0.047*

Sequence divergence was corrected using the K2P model with 2 transitions per transversion.

When using the zebrafish genome as reference, the fastest evolving sequences may not find their homologous counterparts due to the distant evolutionary relationship between the zebrafish and our non-model catfish. Thus, performing the same analysis yet using an evolutionary closer reference - our EST database of the catfish *Hypostomus *gr. *plecostomus *- may allow a better understanding of the efficiency of ISSH method. The sequence divergence comparisons (Table 1) show again a systematic and significant enrichment in fast evolving sequences in the enriched library as compared to the control library. The difference between the two libraries is generally higher than when using the zebrafish as reference. This is likely explained by the inclusion of a set of faster evolving genes which can now find their homologues in the evolutionary closer *Hypostomus *reference.

### Characteristics of the sequences retained by the ISSH method

**Figure 2:**
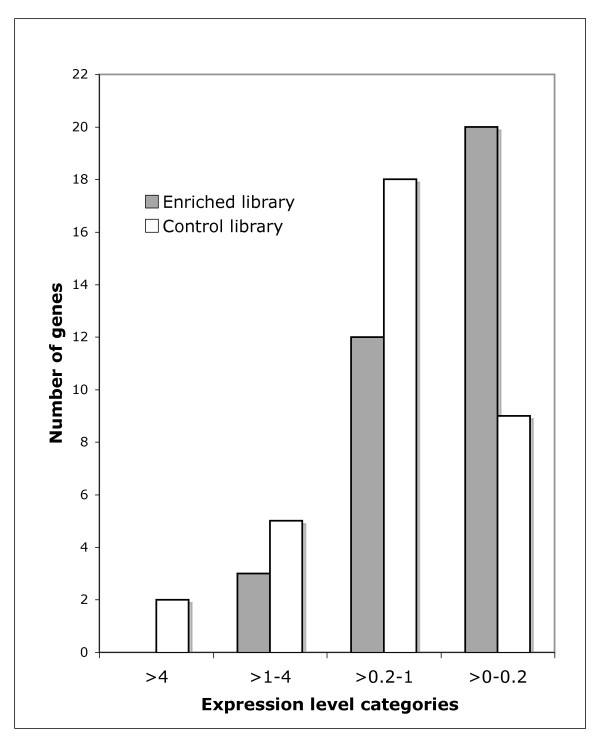
**Number of annotated contigs per category of gene expression level for the enriched and control libraries**. Using Unigene database information, gene expression level is calculated as the number of ESTs of the gene under consideration in the studied tissue divided by the total ESTs of the tissue library, multiplied by 10'000.

In order to better assess the usefulness of the ISSH method we annotated the contigs of the enriched and control libraries according to the information collected from their translated best Blast hit in the Swissprot/Uniprot database with a minimum threshold of E-score ≤ 10e-8. In this way only 60 contigs were characterized in the enriched library (2.5% of all contigs) and 39 in the control library (3.1% of all contigs). The analysis of the annotated sequences will serve to test three predictions that have to be fulfilled if the method achieves its goal. First, as mitochondrial genes evolve significantly faster than the vast majority of nuclear genes they should be more numerous in the enriched library than in the control. The results indicate that mitochondrial genes represent 22.5% versus 8.6% of the annotated contigs of the enriched and control libraries, respectively, fulfilling the prediction. The second expectation concerns the overall correlation between the expression level and gene sequence conservation, where conserved genes are generally expressed at higher rates than fast evolving genes [24]. The expression level of the annotated contigs were approximated by using Unigene database information on the expression level of their orthologous genes in nervous system tissues of the zebrafish or, alternatively, of the mouse or human when the data was not available. Annotated contigs were classified into four categories of gene expression levels (Figure 2). We discarded here the mitochondrial genes which are fast evolving yet possess a high expression level. As expected, the enriched library is essentially composed by genes belonging to the category with the lowest expression-level (58% of the total versus 26% in the control library). The enriched library also shows a depletion of genes in the highly expressed gene categories, which are generally the more conserved ones. The third prediction refers to the observation that genes with tissue-specific expression evolve generally faster than genes with ubiquitous expression, a category in which most housekeeping genes are found [25,26]. As predicted, our method resulted in an enrichment of non-ubiquitously expressed genes totaling 57% of the annotated contigs of the enriched library versus 35% in the control library. The non-ubiquitously expressed genes are defined as those expressed in less than four tissues according to Unigene expression information.

In an attempt to better characterize the fast evolving transcripts isolated in *Ancistrus temminckii *and which show a putative orthologous sequence in the *Hypostomus *gr. *plecostomus *EST dataset, we search for a tentative annotation by Blastn comparisons against the mRNA reference sequences (mRNA refseq) database of NCBI, limited to teleost sequences (a threshold of E < 1e-8 was used). In this way, 26 transcripts present in the catfishes *Ancistrus *and *Hypostomus*, as well as in the teleost mRNA refseq database were annotated and their sequence divergence was calculated based on the overlapping region of the sequence alignment, allowing a direct comparison (Table 2). The tentatively annotated transcript fragments show an overall high sequence divergence between the two Hypostominae catfishes (mean = 0.28 ± 0.09), not much different from the divergence between *Ancistrus *and the closest teleost orthologous sequence of the mRNA refseq database (mean = 0.39 ± 0.08), which do not include catfish sequences. Interestingly, about half of the transcript fragments encompasses coding sequence (cds) (Table 2). To estimate whether the sequence divergence is higher than in recognized fast evolving coding and non-coding DNA markers, we calculated the sequence divergence of the mitochondrial cytochrome oxydase I gene (COI), used by the Barcode of Life Initiative for characterizing species http://www.dnabarcodes.org/, and the two introns of the reticulon 4 (RTN4) gene, used to infer fish phylogenies at the specific level [2]. Most of the tentatively annotated transcripts display higher sequence divergence between *Ancistrus temminckii *and *Hypostomus *gr. *plecostomus*, as compared to the two reference markers (Table 2), validating once again the ISSH method. Likewise, most of the transcripts show higher sequence divergence between *Ancistrus temminckii *and *Danio rerio *as compared to the COI sequence. However, the intronic sequences of the RTN4 are much more divergent than the annotated transcripts. This is likely explained by the frequent insertion/deletion events in non-coding sequences and which enhance drastically the sequence divergence between distantly related species.

**Table 2:** Tentatively annotated fast evolving transcript fragments and their sequence divergence as compared to the closest ortholog in the teleost mRNA refseq database and in the *Hypostomus *gr. *plecostomus *EST dataset.

contig	mRNA refseq annotation according to closest teleost ortholog	Species	Cds/UTR	*A.temminckii *vs closest teleost ortholog in mRNA refseq	*A. temminckii *vs *H*. gr. *plecostomus*
342	zgc:175146	Dr	3'UTR	0.4411	0.2990
435	interferon regulatory factor 6 (irf6)	Dr	3'UTR	0.4824	0.3485
478	NADH dehydrogenase (ubiquinone) 1 alpha subcomplex, assembly factor 2 (ndufaf2), nuclear gene encoding mitochondrial protein	Dr	3'UTR	0.2680	0.2531
597	similar to porcupine homolog (LOC100148644)	Dr	3'UTR	0.4824	0.3485
605	zgc:158374	Dr	cds	0.4411	0.2833
710	single-minded homolog 2 (sim2)	Dr	3'UTR	0.4411	0.3151
785	similar to pol polyprotein (LOC796496)	Dr	cds	0.3839	0.1324
809	similar to ORF1-encoded protein (LOC100004717)	Dr	5'UTR	0.4824	0.3839
1137	zgc:56382	Dr	cds	0.3485	0.2680
1451	RMD5 homolog B (rmd5b)	Ss	5'UTR	0.5042	0.1324
1479	similar to ORF1-encoded protein (LOC100004764)	Dr	3'UTR	0.2531	0.1573
1492	wu:fc33e05	Dr	3'UTR	0.3151	0.2680
1565	hypothetical LOC570897	Dr	cds	0.3316	0.2385
1614	ras-related C3 botulinum toxin substrate 1 (rho family, small GTP binding protein Rac1) like (rac1l)	Dr	3'UTR	0.2833	0.2990
1694	similar to NLR family, pyrin domain containing 3 (LOC100002061)	Dr	3'UTR	0.3839	0.4024
1695	monoacylglycerol O-acyltransferase 2	Dr	3'UTR	0.3485	0.2531
1782	similar to G protein-coupled receptor 128 (LOC100148710)	Dr	cds	0.3839	0.2531
1819	similar to Uromodulin precursor (Tamm-Horsfall urinary glycoprotein) (THP) (LOC100007639)	Dr	cds	0.4824	0.4024
1902	hypothetical protein LOC100150258	Dr	cds	0.3839	0.1324
1905	si:dkeyp-27b10.2	Dr	cds	0.3485	0.2531
2016	zgc:64076	Dr	3'UTR	0.2680	0.1702
2029	zgc:85811	Dr	cds	0.3151	0.1833
2066	similar to CG6639 CG6639-PA (LOC100000002)	Dr	cds	0.4824	0.3151
2085	hypothetical protein LOC100149782	Dr	cds	0.2990	0.2103
2225	zgc:158374	Dr	cds	0.4614	0.4214
2342	similar to zymogen granule membrane glycoprotein 2 (LOC100005977)	Dr	cds	0.4614	0.4411
	Reference fast evolving sequences			*A. brevipinnis *vs *Danio rerio*	*A. brevipinnis *vs *H. boulengeri*
	cytochrome oxidase subunit I (COI)		cds	0.239	0.145
				*A. cirrhosus *vs *Danio rerio*	*A. cirrhosus *vs *H. boulengeri*
	reticulon 4 (RTN4) introns 1 & 2		introns	0.748	0.170

Sequence divergence was calculated using the alignment region with sequence in all species compared. Divergences were corrected using the K2P model with 2 transitions per transversion. The lowest part of the table presents the sequence divergence of two published fast evolving markers used for characterizing species or genera. Dr: *Danio rerio*; Ss:

*Salmo salar*; *A*.: *Ancistrus*; *H*.:*Hypostomus*.

We emphasize that the sequences of the transcripts annotated using the mRNA refseq database likely represent the most conserved regions of the isolated transcripts dataset, as faster evolving regions will not find their sequence counterparts in the refseq database, which comprises no closely related catfish sequences.

## Discussion

The isolation of fast evolving genes can be easily accomplished on model organisms for which abundant genomic and transcriptomic knowledge exist. Bioinformatic routines and experimental procedures (micro-array technology) are available for this purpose. At present, however, there is no efficient method for doing so in non-model organisms. The ISSH method presented here is a fast and cost-effective procedure for enriching a cDNA library in fast evolving genes. The various tests we have performed resulted in a convincing demonstration of the efficiency of our method. We have shown that the overall sequence divergence was significantly increased in the enriched library as compared to the control when blasting these libraries against the zebrafish genome or against our *Hypostomus *catfish EST library. Moreover, the results of the ISSH method fulfilled the three predictions made upon the knowledge of general properties of fast versus slowly evolving genes. Briefly, the enriched library displayed (1) a higher proportion of fast evolving mitochondrial genes, (2) a higher fraction of genes with low expression level, and (3) proportionally more non-ubiquitously expressed genes. Furthermore, the fast evolving transcripts with orthologous sequences in the two catfish species and in the mRNA refseq fish database displayed generally higher sequence divergence than recognized fast evolving DNA markers.

**Figure 3:**
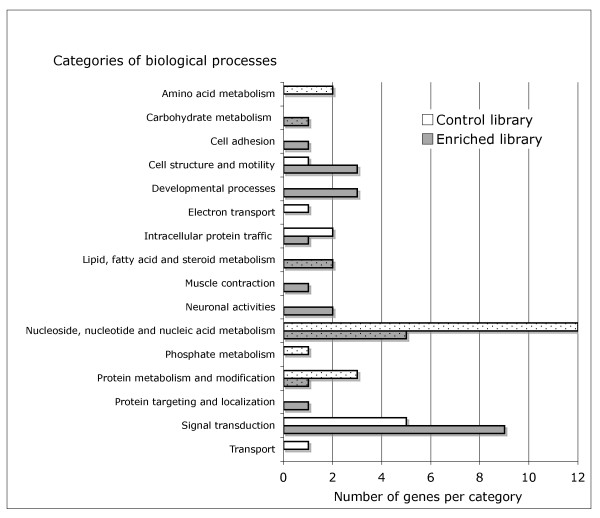
**Gene ontology classification of the fraction of annotated transcripts belonging to the library enriched in fast evolving genes and the control library**. Only the major categories of biological processes are used, according to Panther database. Dotted bars indicate biological processes involved in metabolism.

A complementary support of these results comes from the Gene Ontology (GO) classification. The GO annotation (Figure 3) showed that genes involved in metabolic processes were less abundant in the enriched library than in the control (30% versus 64% of all annotated contigs in the enriched and control libraries, respectively). This is consistent with the observation that the set of housekeeping genes is generally rich in metabolic genes, for example in human [27]. Besides indicating that the enriched library is depleted in housekeeping genes, the GO annotation also shows that this library is enriched in nervous system tissue-specific genes (Neuronal activity, Figure 3), highlighting once more the ability of the method in isolating genes with tissue-specific expression that generally evolve faster than ubiquitously expressed genes.

The proportion of annotated contigs via Uniprot/Swissprot comparisons is rather small, particularly in the enriched library. This can be explained firstly by the relatively poor representation of fish genes in the Uniprot/Swissprot database combined with the likely high sequence divergence between the genes of the non-fish organism in the database and our catfish. Secondly, not all contigs may contain coding sequence; they may be composed mainly of UTR sequence. However, the enriched library shows no marked bias toward UTR sequences, which evolved generally faster than their contiguous coding sequences. Indeed, about 68% of contigs longer that 240 bp display putative open reading frames (ORF) longer than 80 aa (criterion of the H-invitational annotation project), and 51% of contigs longer that 300 bp display putative ORFs longer than 100 aa (criterion of the Functional Annotation of Mouse (FANTOM) project), which corresponds to four and five times the calculated sequence length without stop codons in non-coding sequence using the same base frequencies, respectively. Similar proportions are observed in the control library (70% and 54%, respectively) indicating no strong enrichment in UTR sequences. Furthermore, a significant part of the isolated transcripts may be non-coding RNAs. It has been shown, for instance, that non-coding RNAs constitute more than half of the mammalian transcriptome [28]. As the annotation of the isolated fast evolving transcripts is difficult due to the lack of sequence similarity with distant reference species, we are unable for the moment to assess the proportion of fast evolving non-coding RNAs in our dataset.

Our method has the advantage of being theoretically very versatile in terms of evolutionary divergence relating the species of interest and its selector species. The faster evolving genes will already show detectable sequence divergence between closely related species while using a more distant selector species will allow the isolation of a wider set of fast evolving genes. Likewise, one can modify the hybridization temperature to fine-tune the degree of sequence divergence one is looking for between the species of interest and the selector species. Our method may also be applicable for screening intra-species gene-associated polymorphism. Only in that objective the ISSH method may be compared with the In-Gel Competitive Reassociation and EST Array Hybridization method [29], which exploits the property that the vast majority of RFLP fragments between two strains or populations share the same electrophoretic size. Deviation from this property generates false positives and, therefore, the method of Gotoh and Oishi (2003) looses its interest if more distantly related groups are used.

Interestingly, the ISSH method can also be used for isolating the fraction of highly conserved genes between species. This is achieved by rescuing the fraction of ss cDNA that disanneal only at very stringent conditions, which guarantees an almost perfect complementation between the probed and selector pool of transcripts. Moreover, the species from which the selector pool of mRNA is extracted may be selected in order to increase the level of conservation of the enriched cDNA library: the more evolutionary distant the selector species will be, the more conserved the isolated transcripts will be.

The ISSH method is not linked to a specific sequencing technology. In this study we used the long-read 454 FLX technology (Roche) to ensure a minimum sequence length for downstream sequence analyses. However, this argument is currently less valid as the Illumina short-read sequencing technology, which produces many more reads at a lower cost per base, has been recently shown to be useful and accurate in *de novo *transcriptome assembly of non-model organisms [30]. Traditional Sanger sequencing can also be used providing that the PCR amplified fast evolving transcripts are cloned before sequencing.

## Conclusions

We demonstrated that the ISSH method efficiently enriches a cDNA library in fast evolving genes. As this new method does not rely on the previous knowledge of sequence information, it can be performed on every non-model organism, and is therefore of wide use. Although the improvements and reduced cost of next-generation sequencing technologies may lead to ever more complete transcriptomes assemblies, and may have the potential to be used for identifying fast evolving transcripts with bioinformatic tools, the ISSH method will still have and interesting role to play. First, the ISSH method is inexpensive, of little labor, and leads directly to the set of transcripts of interest. Second, as the fast evolving genes are often expressed at low level, they may be hard to retrieve using next-generation sequencing technologies unless very deep sequence coverage is performed, at high cost. Therefore, the ISSH method opens new possibilities in screening transcriptomes in search of genes involved in lineage specific processes and traits, a field of growing interest in evolutionary biology.

## Methods

### RNA extraction and preparation of the control library

Total RNA was extracted from fresh brain tissue of *Ancistrus temminckii *(probed species) and its close relative *Ancistrus dolichopterus *(selector species) using TRIzol reagent (Gibco). We also extracted total RNA from our catfish outgroup reference *Hypostomus *gr. *plecostomus*. After quantification and quality verification of the total RNA, mRNA was isolated using the mRNA Isolation Kit (Roche Diagnostics). The SuperScript double-stranded cDNA synthesis kit (Invitrogen) was used to prepare the brain control library of *Ancistrus temminckii *and also the outgroup reference *Hypostomus *gr. *plecostomus*, starting with 1 μg of brain mRNA and the option of oligo(dT) anchor priming for the first strand synthesis step.

### The ISSH protocol

The selector pool of mRNA, extracted in this work from *Ancistrus dolichopterus*, is biotinylated to allow subsequent separation by magnetic particles coated with streptavidin. Biotinylation of 5 μg mRNA was done using the BIO-ULS labeling kit (Kreatech); the final volume was reduce to 7 μl using a Speedvac concentrator. The probed pool of mRNAs extracted from the species of interest *Ancistrus temminckii *is reverse-transcribed into ss cDNA using a short-tailed random hexamer primer (5'-AGGA-(N)6-3'). We used 1 μg of mRNA (one fifth of the selector's mRNA amount) and 200 ng of the short-tailed random primer in a total volume of 12 μl. The reverse transcription was performed using the SuperScript II RT (Invitrogen) following the manufacturer's protocol for random priming; the final volume was 20 μl. The RNA template is destroyed by alkaline hydrolysis (0.35 N NaOH; 0.35 M EDTA) at 65°C for 15 min. The solution is then neutralized with 0.35 N HCl and first strand cDNAs are purified using the Mini Elute PCR Purification Kit (Qiagen) following the manufacturer's protocol but with an additional washing step and two rounds of elution. The final volume was reduced to 7 μl using a Speedvac concentrator.

### Inter-Specific Selective Hybridization

The pool of biotinylated selector mRNA (7 μl) and the pool of first strand cDNA of the species of interest (7 μl) are mixed and the total volume is adjusted to 15 μl. An equal volume (15 μl) of 2× hybridization buffer is added (10 mM EDTA pH8, 1.5 M NaCl, 40 mM sodium phosphate buffer pH 7.2, 10× Denhardt's, 0.2% SDS). The solution is heated at 90°C for 2 min and quickly placed in a rotary shaker located inside a preheated hybridization oven at 55°C. The hybridization is carried on during 60 hours at 55°C. At the end of the hybridization step, 75 μl of NaCl 1 M is added to the hybridization mixture, which is kept at RT.

### Separation of the fraction enriched in fast evolving cDNAs

The selector-probed hybridization mix is sequentially denatured to separate two fractions of cDNAs with increasing denaturation stringencies, the first fraction containing the non-hybridized or non-specifically hybridized probed cDNAs and the second fraction is the one enriched in fast evolving transcripts. First, streptavidin magnetic particles (Roche Diagnostics) are prepared according to the manufacturer's instructions (1200 μg) and resuspended in 100 μl of TEN 1000 buffer. The hybridization mixture is then transferred in to the tube containing the streptavidin magnetic particles and placed in a rotary shaker for 45 min at RT. In this step the biotinylated selector mRNAs, which may be hybridized or not with a complementary probed ss cDNA, are linked to the streptavidin magnetic particles. The non-hybridized probed cDNAs are discarded by placing the tube in a magnetic separator (Qiagen) and by removing the supernatant. The magnetic particles with their attached molecules are washed three times at 55°C for 15 min, in 600 μl of preheated 5× SSC, then resuspended in 50 μl of 0.1× SSC and incubated at 65°C for 15 min. In this last step the fast evolving probed cDNAs will disanneal from their selector counterpart and this fraction of interest is recovered in the supernatant after a magnetic separation. This step is repeated once. The fraction enriched in fast evolving cDNAs is purified by ethanol precipitation in presence of ammonium acetate and glycogen. The pellet is rinsed once in 70% ethanol and resuspended in 20 μl water.

### Second strand synthesis and adapter ligation

The ss cDNAs are transformed into double stranded (ds) cDNAs using short-tailed random hexamer primers (CCAC-(N)6) and the DNA polymerase I Klenow fragment (Promega), according to the manufacturer's random priming protocol. cDNAs are then blunt ended using T4 DNA Polymerase (Promega), extracted with phenol/chloroform/isoamylalcohol (25:24:1) and recovered by ethanol precipitation with ammonium acetate. Double strand EcoRI adapters (Invitrogen) are ligated to the ds cDNA ends according to the manufacturer's instructions. The final volume is adjusted to 100 μl with water and the cDNAs are purified using the High Pure PCR Product Purification Kit (Roche Diagnostics).

### PCR amplification of the fraction enriched in fast evolving cDNAs

A first PCR amplification is performed using a single primer (5'-GTCGACGCGGCCGCGAATT-3') targeted toward the EcoRI adapter ligated at both ends. The PCR reaction is done in 50 μl final volume with 10 μl of cDNA as template and with the following profile: 1 min initial denaturation at 94°C followed by 35 cycles with 30 s at 94°C, 30 s at 62°C, 2.5 min at 72°C and a final elongation step of 5 min at 72°C. The PCR product is checked on 1,5% agarose gel. A nested PCR is performed using specific primers overlapping the end of the EcoRI adapters and the tails of the two short-tailed random primers used for the synthesis of the first strand and then for the synthesis of the second strand (EcoRI-AGGA: 5'-TCGCGGCCGCGTCGACAGGA-3'; EcoRI-CCAC: 5'-TCGCGGCCGCGTCGACCCAC-3'). The PCR conditions are as described above but the amount of template DNA is adjusted according to the result of the first PCR. The PCR products are checked on 1,5% agarose gel and then purified using the High Pure PCR Product Purification Kit (Roche Diagnostics).

### High-throughput sequencing

For the *Ancistrus *control and the outgroup reference *Hypostomus *gr. *plecostomus*, shotgun DNA libraries were prepared with a starting amount of 4 μg DNA. The mean fragment size was of about 500 bp, obtained using nebulizers and chemicals from the GS DNA Library Preparation Kit (Roche Diagnostics) according to the manufacturer's manual. This step was not needed for the *Ancistrus *library enriched in fast evolving transcripts as the ISSH method results in a PCR product containing fragmented transcripts, generally in the range of 300 to 1000 bp. After DNA purification, the DNA end repair step and the ligation of the barcoding adaptors were performed following established protocols [31]. The adapter-ligated DNA from each of the three libraries were pooled and prepared for the 454 sequencing according to standard protocols [32], using the GS DNA Library Preparation Kit with Titanium reagents, and following the instructions of the GS FLX manuals (Roche Diagnostics). The library was sequenced on one 16th region of a full GS FLX sequencing plate with a prior titration run. Upon completion, sequences were screened for primer concatemers, week signal, poly A/T sequences, and barcodes for assigning them to one of the three samples. The average lengths of the reads were 180 bp. cDNA assemblies were performed with the SeqMan software from DNAStar. The cDNA library enriched in fast evolving genes and the control library *of Ancitrus temminkii *were deposited in the Short Read Archive (SRA) of NCBI under the accession number SRA009346.1

### Blast search and transcript annotation

The Blast search against the zebrafish sequences of all the EMBL sub-divisions (Expressed Sequence Tag; High Throughput cDNA sequencing; High Throughput Genome sequencing; mRNA of Standard; Whole Genome Shotgun) were performed on the Vital-IT high-performance computing facility of the Swiss Institute of Bioinformatics http://www.vital-it.ch. We used blast parameter values suitable for comparing divergent sequences (word size = 7; match score = +1; mismatch score = -1; initial penalty for opening a gap = 1; penalty for extending a gap = 2). The local Blast search against our *Hypostomus *gr.* plecostomus *brain EST database was performed using the software blast-2.2.19 developed by NCBI. Perl scripts for parsing the blast outputs were built using Eclipse SDK 3.4.1.

The proportion of contigs with low complexity sequences or sequence repeats was assessed using RepeatMasker open-3.2.8 (Smit, AFA, Hubley, R & Green, P. *RepeatMasker Open-3.0*. 1996-2004; http://www.repeatmasker.org). Transcripts were annotated according to their best Blast hit against Swissprot/Uniprot databases, with a minimal E-score of 10e-8. The translations into the six frames were performed using BCM Search Launcher [33] and blasting was done with Blastp at NCBI. Expected frequency of stop codons in non-coding sequences was calculated by multiplying the three single nucleotide frequencies taken from the sequence data of the corresponding library, and summing the frequency of the three possible stop codons. Gene transcription levels in specific tissues were taken from the Unigene database and are expressed in number of ESTs of the gene under consideration divided by the total ESTs of the tissue library, multiplied by 10'000. Gene ontology classification was performed on Panther (*Protein ANalysis THrough Evolutionary Relationships*; http://www.pantherdb.org), complemented with ontology information given in Uniprot database. We used only the top categories of the classification hierarchy, as given in Panther. Fast evolving transcripts found in *Ancistrus temminckii*, *Hypostomus *gr. *plecostomus *EST, and in the mRNA reference sequences database of NCBI, restricted to the Teleostei (Blastn threshold E-score < 10e-8), were used to asses the sequence divergence. A tentative annotated was given according to the best hit against the mRNA refseq database. For direct comparison purposes, sequence divergence was calculated on the sequence region present in all three taxa. Sequences of the reference fast evolving markers were obtained from GeneBank: *Ancistrus brevipinnis *COI: EU359402; *Hypostomus boulengeri *COI: EU359422; *Danio rerio *complete genome: NC_002333; *Ancistrus cirrhosus *RTN4 introns: EU817562; *Hypostomus boulengeri *RTN4 introns: EU817560. The RTN4 introns from *Danio rerio *were retrieved from Ensembl http://www.ensembl.org/, locus: chromosome: Zv8:1:42092991:42094205:1.

### Data access

Raw read data is available at the Short Read Archive (SRA) of NCBI under the accession number SRA009346.1

No ethical approval was required for this study.

## Authors' contributions

JIMB conceived the project and designed the analyses. JIMB and AF performed the bioinformatic and sequence analyses. JIMB and IB performed the experimental work. The manuscript was prepared by JIMB with assistance from AF and IB. All authors have read and approved the final version of the manuscript.
